# Contraceptive use among sexually active female adolescents in Ethiopia: trends and determinants from national demographic and health surveys

**DOI:** 10.1186/s12978-021-01161-4

**Published:** 2021-05-25

**Authors:** Alemi Kebede Olika, Sena Belina Kitila, Yonas Biratu Terfa, Ayantu Kebede Olika

**Affiliations:** 1 Population and Family Health Department, Faculty of Public Health, Institute of Health Science, Jimma University, Jimma, Ethiopia; 2 School of Nursing and Midwifery, Faculty of Health Sciences, Institute of Health Science, Jimma University, Jimma, Ethiopia; 3 Department of Epidemiology Faculty of Public Health, Institute of Health, Jimma University, Jimma, Ethiopia

**Keywords:** Adolescent, Contraception, Family planning, Sexual and reproductive health and right, Utilization, Ethiopia

## Abstract

**Background:**

Sexual and reproductive health and right of adolescents is a global priority as the reproductive choices made by them have a massive impact on their health, wellbeing, education, and economy. Teenage pregnancy is a public health issue and a demographic challenge in Ethiopia. Increasing access to contraceptive services for sexually active adolescents will prevent pregnancies and related complications. However, little is known about the trends in contraceptive use and its determinants among adolescent girls in Ethiopia. Therefore, this study was designed to examine the trends and factors associated with contraceptive use among sexually active girls aged 15–19 years in Ethiopia by using Ethiopian demographic and health survey data.

**Methods:**

Four Ethiopian demographic and health survey data were used to examine trends of contraceptive methods use. To identify factors associated with contraceptive use, the 2016 Ethiopian demographic and health survey data were used. The data was downloaded from the demographic and health survey program database and extracted for sexually active adolescent girls. Data were weighted for analysis and analyzed using SPSS version 21. Descriptive analysis was used to describe the independent variables of the study. A multivariable logistic regression model was used to identify factors associated with contraceptive use and adjusted odds ratios with 95% confidence interval were presented for significant variables. Variables with a *p*-value less than 0.05 were considered as significantly associated with contraceptive use.

**Results:**

Contraceptive method use had increased significantly from 6.9% in 2000 to 39.6% in 2016 among sexually active adolescent girls in Ethiopia. The odds of contraceptive use were lower among female adolescents who had no formal education (AOR 0.044; 95% CI 0.008–0.231) and attended primary education (AOR 0.101; 95% CI 0.024–0.414). But the odds were higher among adolescents from a wealthy background (AOR 3.662; 95% CI 1.353–9.913) and those who have visited health facilities and were informed about family planning (AOR 3.115; 95% CI 1.385–7.007).

**Conclusion:**

There is an increment in the trend of contraceptive use among sexually active female adolescents in Ethiopia between 2000 and 2016. Significant variations in the use of modern contraception by wealth status, educational level and visited a health facility, and being informed about family planning were observed. Improving the economic and educational status of young women, and provision of information may help in improving contraceptive use in Ethiopia.

## Plain English summary

Adolescent contributes up to 16 per cent of the world’s population. Sexual and reproductive health and right of these populations is a global priority. Teenage pregnancy is a public health issue and a demographic challenge in Ethiopia. A contraceptive method is best for sexually active adolescents to prevent pregnancies and its related complications.

Data for this study are from the four Ethiopian Demographic and Health Surveys [EDHS] conducted in 2000, 2005, 2011, and 2016. Data for sexually active adolescent girls were extracted from the data set.

The trend of contraceptive use among adolescents is increasing from time to time. However, more than three-fifth of the sexually active female adolescents are still not using any type of contraception. There were significant variations in the use of modern contraception by background characteristics of the respondents. In all four rounds of the EDHS, modern contraceptive use was common among adolescents in 18–19 years, who attended secondary and above educational level, lived in urban areas, and was from wealthy family groups.

## Introduction

Adolescence is a critical phase in life and a time of social and biological transition between childhood and adulthood that entails numerous milestones and opportunities, roles, and responsibilities [1–3]. Adolescents are individuals in the age range of 10–19 years. This age group constitutes 16 per cent of the world’s population and is one of the fastest-growing cohorts, and their contribution is vital in achieving several developmental goals [4–6]. Global developmental goals and strategies have recognized the importance of adolescent's health and rights [7–9]. Sexual and reproductive health and rights (SRHR) of these populations is a global priority as the reproductive choices made by them have a massive impact on their health, wellbeing, education, and economy [10, 11].

In low- and middle-income countries (LMICs), unintended pregnancy accounted for half of all pregnancies among girls and more than half of these pregnancies result in abortion [10]. Annually, about 16 unsafe abortions per 1000 occur in LMICs among girls under the age of 20 years [12]. Pregnancy and childbirth complications are the leading cause of death among adolescent girls globally, with a higher proportion in LMICs [13, 14]. Besides, children born to adolescent girls are more likely to have low birth weight [15]. Access to sexual and reproductive health information and services are essential in averting these problems and deaths [1, 3, 11, 16].

Increasing access to contraceptive services for sexually active adolescents will prevent pregnancies and related complications [10]. For instance, the use of modern contraceptives prevented an estimated 308 million unplanned pregnancies in 2017 [11]. If the modern contraceptive method were accessible for adolescents with unmet needs, unintended pregnancies would drop by 59% [10]. Also, expanding the contraceptive method mix can serve as an effective strategy to prevent unsafe abortion [17]. Hence, all adolescents who want to prevent pregnancy should be able to obtain and use contraception [18].

Available evidence shows that sexually active unmarried adolescents do not seek to become pregnant and the married wish not to become pregnant at a young age or wish to delay a second pregnancy [11, 14, 19, 20]. However, only, 40% of adolescents are using an effective contraceptive method in LMICs [10, 21]. Despite having clear needs, adolescents often fail to access contraceptives, and unplanned pregnancies happen despite the best of contraceptive intentions. There are disparities in contraceptive use between different age groups and young people have inadequate access to sexual and reproductive health information and services [2, 3, 22].

In Ethiopia, teenage pregnancy is a public health issue and a demographic challenge. The proportion varies geographically with 15% in rural and 5% in urban in 2016 [22]. Preventing teenage pregnancies and fertility is among the priority issues of the Ethiopian Federal Ministry of Health [23]. Ethiopia's government is implementing youth policies and national adolescent and youth health strategies to support young people in increasing their access to sexual and reproductive health services [24–26].

Though there are primary studies on contraceptive use in Ethiopia [27, 28], there is a lack of evidence concerning trends in contraceptive use and its determinants among girls aged 15–19 years from the data that represent the national level picture. To effectively respond to this growing population's reproductive health needs, it is imperative to understand their contraception practices. Therefore, this study aimed to examine the trends and correlations of contraceptive use among sexually active adolescents in Ethiopia by using the Ethiopian demographic and health survey data. The study's findings will be useful for health planners, policymakers, and developmental partners who are working to improve the health and well-being of adolescents in Ethiopia. Furthermore, it will assist in designing programs and strategies to increase coverage, quality, and equity of adolescent girl’s reproductive health at the country level.

## Methods

### Data source

Data for this study were extracted from the four rounds (2000, 2005, 2011, and 2016) of EDHS. EDHS consists of a sample of households obtained through a two-stage stratified sampling procedure. Regions were stratified into urban and rural areas. Each stratum was clustered into Enumeration areas (EAs) based on the 2007 Ethiopian housing and population census. Samples of EAs were selected independently in each stratum in two stages. The first stage involves randomly selecting clusters, EAs. A sample of households is drawn from a listing of households in each of the sampled clusters at the second stage.

The information about contraceptive use was collected from all non-pregnant, fecund reproductive-age women using a pretested questionnaire. All four rounds of EDHS data were used to describe the trends of contraceptive use among sexually active female adolescents. The 2016 EDHS data were employed to analyze the determinants of contraceptive use. Permission to use the data was granted by the Measure DHS program on April 1, 2019.

### Operational definitions

Sexually active: Those respondents who reported that they have had sexual intercourse, irrespective of their marital status in the last four weeks at the time of interview.

Contraceptive use: Respondents who at the time of interview said they or their partner are using any contraceptive method to delay or avoid becoming pregnant. It was dichotomous denoting users and nonusers of contraceptive methods.

Current use of modern contraception: Current users of modern contraceptives include young women who said that they or their partner are currently using any of the following modern methods of contraception, such as female sterilization, male sterilization, the pill, injectable, intrauterine device, implant, condom, and emergency contraception at the time of the survey.

### Study population and sample size

The study populations were sexually active adolescent girls between 15 and 19 years. During the four rounds of the survey, 3710, 3266, 4009, and 3381 adolescent girls have participated respectively. Of the total adolescent girls who participated, 762, 287, 632, and 504 were sexually active in the last four weeks before data collection and used for analysis in 2000, 2005, 2011, and 2016 respectively.

### Study variables

The dependent variable was contraceptive use. Independent variables include age, educational status, religion, ethnicity, marital status, working currently, wealth status, visited a health facility in the last 12 months before the survey, being informed about family planning (FP) at the health facility, heard information about FP from radio, watch on Television (TV) and read it on newspaper and place of residence.

Some of the study variables were re-coded to suit the purpose of the study while some were used as they are in the original data set. For instance, religious affiliation was re-coded into orthodox Christian, other Christian, Muslim and other, by combining catholic and protestant for other Christian. Wealth status was also re-coded into poor, average, and rich by combining “poorer” and “poor” for poor and “rich” and “richer” for rich. The highest education level was re-coded into no education, primary, and second and above by combining secondary and higher.

### Data analysis

The data was downloaded from the demographic and health survey program database. The data for sexually active adolescent girls were extracted and data cleaning was done before any analysis. The analysis was performed by using IBM SPSS Statistics for Windows, Version 21.0. Armonk, NY: IBM Corp; 2012. Before any statistical analysis, standard EDHS sample weights were applied to account for the unequal probability of selection in the sample and non-response. The recommended procedure on how to weight DHS data in SPSS was followed. The weighting variable used was women's individual sample weight, since the study unit of analysis is women. The weight variable was created by dividing the individual women sample weight variable by 1,000,000.

Descriptive analysis was used to describe the background characteristics of the study participants. Multicollinearity was checked before running logistic regression using variance inflation factor. The maximum value of the variance inflation factor was 1.119, indicating that the absence of multicollinearity. Binary and multiple logistic regressions were employed to identify candidate variables and examine the determinants of contraceptive use respectively. Adjusted odds ratios with 95% confidence interval were presented for significant variables in the final model to estimate the likelihood of contraceptive use among various categories of adolescents. Variables with a *p*-value less than 0.05 were considered as significantly associated with contraceptive use. All figures and tables in the report depict weighted numbers and percentages.

## Results

### Trends in contraceptive use

**Fig. 1:**
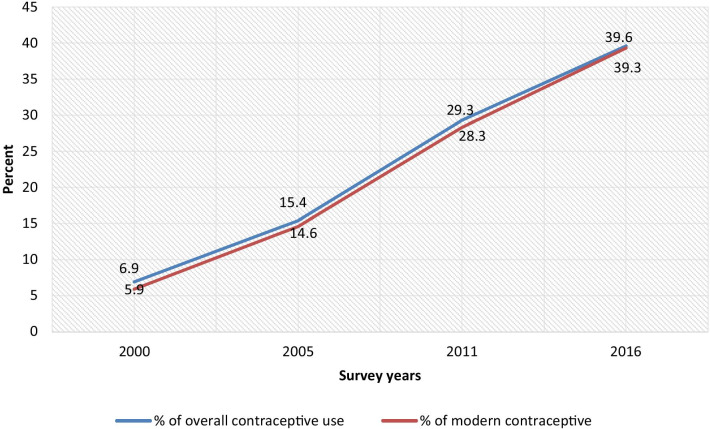
Trends in use of modern contraceptives among sexually active adolescent, Ethiopia DHS 2000–2016

The trends of contraceptive use among sexually active female adolescents increased from 6.9 per cent in 2000 to 39.6 per cent in 2016. The proportion of modern contraceptive use increased by 8.7 per cent between 2000 and 2005 and nearly doubled between 2005 and 2011. Modern contraception use continuously increased between the years 2005 and 2016 and, more than 10 per cent increment was observed between 2011 and 2016 (Fig. 1).

### Disparities in contraceptive use by background characteristics of respondents

**Fig. 2:**
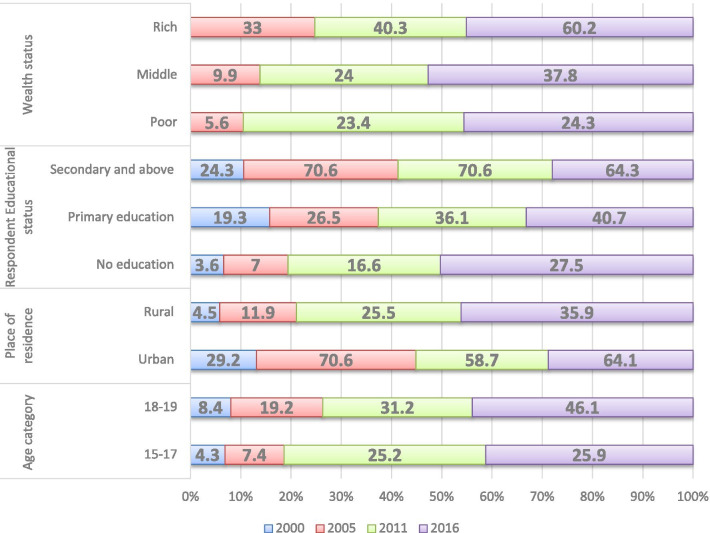
Differentials in contraceptive use among sexually active adolescent by background characteristics, Ethiopia DHS 2000–2016

The proportion of contraceptive use varies significantly with age, place of residence, educational level, and household wealth status of study subjects. In all four rounds of the EDHS, modern contraceptive use was common among adolescents in 18–19 years, who attended secondary and above educational level, lived in urban areas, and was from wealthy family groups. Between 2005 and 2011, the proportion of girls with secondary and above education and using modern contraception methods did not change whereas it declined by 6.3 per cent between 2011 and 2016 (Fig. 2).

### Modern contraceptive utilization trends by method mix

**Fig. 3:**
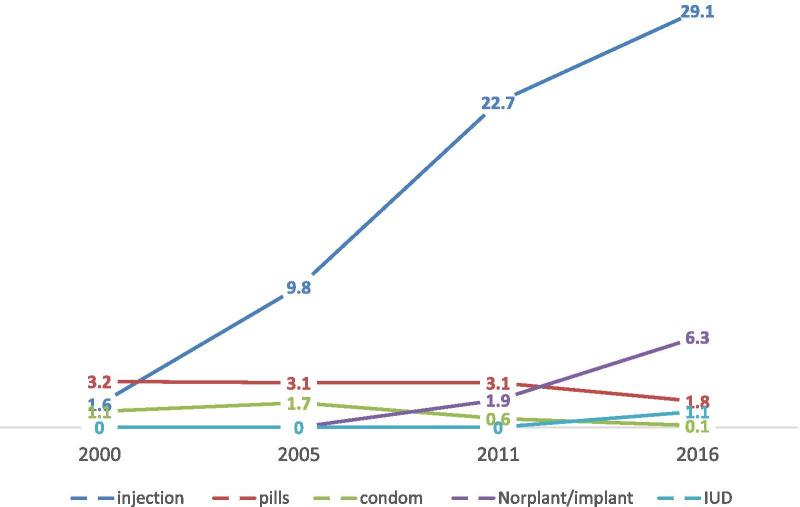
Trends in method mix contraceptive use among sexually active adolescent, Ethiopia DHS 2000–2016

The trends in the mix of currently used modern methods indicated promising improvement. The share of the long-acting reversible contraceptive methods such as Norplant/implants among sexually active adolescent girls meaningfully increased by more than 6 percentage between 2000 to 2016. Intrauterine device (IUD) use did not increase as compared to implants. Utilization of Implants increased by 6.3 per cent, whereas only 1.1 per cent increment was recorded for IUD in 2016. Among short-term methods, the share of injection contraceptive method had been considerably increased by more than 25 per cent from the year 2000 to 2016. The use of condoms had significantly declined between the years 2005 and 2016 from 1.7 to 0.1% (Fig. 3).

## Contraceptive use and its determinants from 2016 EDHS data

### Characteristics of sexually active adolescent girls from EDHS 2016

A total of 504 sexually active adolescent girls were considered for this study. The mean age of the respondents was 17.73 years (SD ± 1.124). The mean ages at first cohabitation and first sexual intercourse were 15.59 years (SD ± 1.734) and 15.63 years (SD ± 1.751) years respectively. Nearly half (225, 47.3%) of adolescent girls cohabited at age 15 years or less.

Three-fifth (60.6%) of the sexually active adolescent girls had attained primary education and 440 (87.3%) were rural residents. While the majority (458, 91.0%) of study subjects were married, and 125 (24.8%) of them were from the low socioeconomic group. Slightly more than two two-fifth (41.9%) and 199 (39.5%) of the girls were Muslims and Oromo by their religion and ethnicity respectively. More than three-fourth (79.7%) of respondents were not working at the time of the survey (Table 1).

**Table 1:** Distribution of sexually active female adolescents age 15–19 by their background characteristics, Ethiopia DHS 2016

Variable		Frequency	Percent
Age category	15–17	161	32.0
	18–19	343	68.0
Education of respondent	No education	143	28.4
	Primary	305	60.6
	Secondary and above	56	11.0
Place of residence	Urban	64	12.7
	Rural	440	87.3
Marital status	Married	458	91.0
	Never married	23	4.5
	Divorced/separated	23	4.5
Wealth index	Poor	230	45.6
	Average	97	19.3
	Rich	177	35.1
Religion	Muslim	211	41.9
	Orthodox Christian	201	39.9
	Other Christian	90	17.9
	Other	2	0.3
Ethnicity	Oromo	199	39.5
	Amhara	155	30.7
	Tigraie	34	6.8
	Sidama	22	4.4
	Somalie	17	3.3
	Walaita	12	2.4
	Gamo	10	1.9
	Guragie	8	1.5
	Other	48	9.4
Respondent occupation	Not working	300	59.6
	Agriculture	102	20.3
	Sales	57	11.3
	Others*	45	8.8
Currently working	No	402	79.7
	Yes	102	20.3
Husband occupation(N = 458)	Not working	35	7.6
	Agriculture	301	65.8
	sales	33	7.2
	Professional/technician	16	3.4
	Others^+^	74	16.1
Husband education(N = 458)	No education	147	32
	Primary	220	48.1
	Secondary and above	91	19.9

*Professional/managerial/technical, services, skilled and unskilled manual

^+^Skilled and unskilled manual, services, I don’t know

### Awareness and knowledge about fertility and contraceptive methods

Among sexually active adolescent girls, almost all (98%) of them had knowledge about contraceptive methods. Only 18.1, 13.4, and 4.2 per cent of girls reported that they had heard about FP messages on radio, watched on television, and read it in newspaper/magazine respectively in the last few months before the survey. More than three-fourth (79.9%) of girls reported that field workers did not visit them in the last 12 months before the survey. Out of those visited by fieldworkers, only 9.9% of fieldworkers talked about FP. Additionally, 77 (15.3%) of girls were informed about FP during their health facility visit in the last 12 months (Table 2).

**Table 2:** Distribution of adolescents age 15–19 by their exposure to family planning information, knowledge about fertility and contraceptive method, Ethiopia DHS 2016

Variables		Frequency	Percent
Heard family planning message on radio on last few months	Yes	91	18.1
	No	413	81.9
Watch family planning messages on TV on last few months	Yes	68	13.4
	No	436	86.6
Read about family planning messages on newspaper/magazine last few months	Yes	21	4.2
	No	483	95.8
Received family planning text message on mobile phone	Yes	5	1.1
	No	498	98.9
Visited by field worker in the last 12 months	Yes	101	20.1
	No	402	79.9
Field worker talk about family planning	Yes	50	9.9
	No	51	10.2
Visited health facility in the last 12 months	Yes	211	42.0
	No	292	58.0
Told about family planning in the health facility	Yes	77	15.3
	No	135	26.7
Knowledge of ovulatory period	During her period	37	7.3
	After period ended	148	29.4
	Middle of the cycle	76	15.0
	Before period begins	33	6.5
	At any time	116	23.0
	Do not know	95	18.8
Knowledge about any contraceptive method	Knows no method	7	1.3
	Knows only traditional method	4	0.7
	Knows modern method	494	98.0
Number of living children	No child	308	61.1
	One child	173	34.4
	Two and more	22	4.5
Decision maker on contraceptive use	Mainly respondent	34	19.7
	Mainly husband	3	1.9
	Joint decision	133	78.4

### Factors associated with contraceptive use among sexually active adolescents

In binary logistic regression, eleven variables had a significant relationship with adolescent girls’ contraceptive use. These include respondent’s and partner’s educational and occupational status, current working status, wealth status, visited by a field worker in the last 12 months, informed about FP at a health facility, place of residence, heard about FP messages on radio, and watched on television in the last few months were considered for multivariate analysis.

The odds of contraceptive use were nearly 96% (AOR 0.044; 95% CI 0.008–0.231) and 90% (AOR 0.101; 95%CI 0.024–0.414) less likely among sexually active female adolescent who had no formal education and primary education respectively as compared to those who had attained secondary and higher education. Adolescents in a rich wealthy background status were three-times more likely to use contraceptive methods than their counterparts (AOR 3.662; 95% CI 1.353–9.913). The odds were three-times among respondents who had visited health facilities and informed about FP as compared to those uninformed about FP during their visit (AOR 3.115; 95% CI 1.385–7.007) (Table 3).

**Table 3:** Bivariate and multivariate logistic regression model showing predictors of contraceptive use among sexually active adolescents, 2016 EDHS

Variables and its category		COR [95% CI]	AOR [95% CI]
Adolescent age category	15–17	.408 (.270, .616)	.524 (.218, 1.260)
	18–19	1	1
Place of residence	Urban	3.256 (1.882, 5.632)	0.692 (.113, 4.238)
	Rural	1	1
Heard family planning on radio last few months	Yes	2.365 (1.491, 3.750)	1.046 (.405, 2.699)
	No	1	1
Heard family planning on TV last few months	Yes	3.323 (1.943, 5.682)	0.374 (.093, 1.506)
	No	1	1
Told about FP at health facility	Yes	3.083 (1.721, 5.523)	3.115 (1.385, 7.007)*
	No	1	1
Husband education	No education	.422 (.247, .722)	1.083 (.320, 3.671)
	Primary	.445 (.271, .731)	0.330 (.107, 1.015)
	Secondary & above	1	1
Wealth index	Poor	1	1
	Average	1.886 (1.135, 3.134)	0.424 (.130, 1.380)
	Rich	4.701 (3.070, 7.198)	3.662 (1.353, 9.913)*
Respondent occupation	Not working	1	1
	Agriculture	1.407 (.891, 2.222)	1.841 (.632, 5.359)
	Sales	2.730 (1.447, 5.154)	2.599 (.387, 17.455)
	Others (skilled, unskilled manual)	1.048 (.577, 1.902)	0.468 (.085, 2.587)
Respondent education	No education	.206 (.106, .399)	0.044 (.008, .231)*
	Primary	.369 (.203, .669)	0.101 (.024, .414)*
	Secondary & above	1	1
Currently working	Yes	1.797 (1.160, 2.784)	2.622 (.660, 10.412)
	No	1	1

*Significant value at 0.05

## Discussion

This study showed that more than nine out of ten adolescents were married. This showed the practice of very early marriage and early sexual activities among adolescent girls. Early marriage often results from the traditional and cultural family values that justify control over women’s sexuality and fertility [29–31]. Such practices have a direct impact on girls' education and future career [32]. It has also a negative consequence on the economic development of nations in addition to causing a significant health risk both to a girl, and her baby [33]. This was due to an extended time that the girls spend in childbearing years that cause an increase in fertility and population growth. The evidence also indicated that in the marriage union, the frequency of sexual activity is higher than in those who are not, hence in the absence of contraception there is a greater likelihood of occurrence of pregnancy [29].

During the last two decades, a growing trend was observed in the use of contraceptives by adolescent girls in Ethiopia. Contraceptive use among sexually active girls increased by six-folds between 2005 to 2016 EDHS. This may be attributed to the implementation of several interventions such as youth-friendly health services and innovative health extension program that brings health services including FP to the communities’ home [34, 35]. There is also a national political commitment to FP in Ethiopia, governments and nongovernmental organizations have increased resource allocations for contraceptive security and delivery [29]. Further, the provision of short-term contraception methods by the private sectors may have also played an important role in increasing young women’s access to contraceptive services in Ethiopia [30].

The proportion of sexually active female adolescent contraceptive users relying on the IUD and implants increased substantially from no reported users in 2000 to 1.1% and 6.3% in 2016 respectively. However, IUD use did not increase compared to implants. This may be because starting from 2009 insertion of the implant was cascaded to the health post level, and training was given to the health extension workers on the provision of the service [36]. Despite the progress that has been achieved, a considerable number of sexually active adolescent girls use short-acting methods, especially injectable, which has a failure rate compared to long-acting and reversible contraceptive methods. The low uptake of long-acting and reversible contraceptive methods may be due to barriers such as lack of availability, fear, misconceptions, and provider bias on the provision of long-acting methods for adolescents [37, 38].

Trends of injectables rose from 1.6 to 29.1%, while the patterns observed in condoms and pill declined from 1.1 to 0.1% and 3.2 to 1.8% respectively between 2000 and 2016. This finding indicated that adolescent girls appear to be shifting away from condoms and pills, and choosing injectable contraceptives. This finding is comparable with a study from Kenya and Rwanda, where, injectable contraceptives have been consistently dominant among women aged 15–24 years [39]. A possible reason could be due to their fertility intention. Adolescent girl intends to delay or space births for a short period which might explain their preference for short-acting methods that are easier to start and stop as needed [40].

Although there are visible changes in the trend of contraceptive use and knowledge of contraception was almost universal, still more than three-fifths of sexually active female adolescents are not using the contraception according to 2016 EDHS. This finding was slightly lower than the report from LMICs where 42–68% of sexually active adolescent females in all the Latin American countries (except Guatemala and Haiti) and in Bangladesh, Indonesia, Kazakhstan, and Turkey were currently using contraceptives [40]. However, it is higher than the results from the African countries where contraceptive prevalence was 20–35%, except in Namibia in which it reached at least 40% [40]. This finding was also slightly higher than Zimbabwe and Malawi's study, where 35% and 33% of adolescents use a contraceptive, respectively [39].

There were significant variations in the use of contraception by background characteristics of adolescent girls in Ethiopia. This study revealed that respondent’s education was an independent predictor for contraceptive use. This was similar to a study conducted in Nigeria and Burkina Faso where contraceptive use among adolescents who attended secondary/above education was higher than those who had completed primary education [28]. Similarly, in Ghana, the odds of contraceptive use were higher among educated female adolescents [41]. Further, low contraceptive use among illiterate female adolescents was reported in Bangladesh [42]. This may be because educated girls are more likely to appreciate the dividend that contraceptive use has on their lives. Also, they may have a plan to pursue the highest career within their education as a result; they want to delay their childbearing time.

The likelihood of contraceptive use increased significantly with the increase in household economic status. This finding was in line with a report from three African countries: Nigeria, Burkina Faso, and Ethiopia. Across all these countries, there is a significant equity gap in modern contraception use because of the wealth status [28]. Likewise, another study in Ethiopia showed that women from richer households were more likely to use contraception [27]. This could be because, most of the small resources obtained from the petty jobs done by women, and their spouses in poor households are diverted for taking care of the family and less is shifted to the health of the mothers. Hence, women from poor households refused the service as they encountered difficulties to cover direct and indirect costs incurred in seeking the services [43].

Further, adolescents who had been told about FP during a health facility visit used contraception more than those who were not told about FP. The existing literature indicated that female adolescent's access to family planning information via different sources increases the use of modern contraceptive methods. For instance, in Nigeria hearing about family planning on mass media was associated with the use of modern contraceptives [44]. Also, being visited by a community health worker resulted in an inclination for modern contraceptive methods [45]. Access to information plays a significant role in the use of contraception as it can raise an individual’s awareness, and influence their attitude, and could guide them to make an informed decision to use the services. However, in the present study, only 15% of those who visit health facilities are told about contraception that indicated many sexually active adolescent girls miss out on this information.

A systematic review conducted in 2011 and updated in 2016, on youth-friendly family planning services for young people indicated the importance that young people place on receiving comprehensive, client-centered family planning counseling [35]. However, several factors were identified as barriers to the delivery of effective contraceptive counseling and care for adolescents. For instance, in Latin America, many consider adolescent use of contraception to be socially unacceptable [46]. As there were significant associations between FP counseling with contraceptive initiation and continuation, health care provider skills in the counseling, and provision of contraception for an adolescent is, therefore, needed to be emphasized [47].

The main strength of this study was the use of nationally representative data. The study has also some limitations, the small sample sizes that contributed to a bit wider confidence level for some variables. Possibility of social desirability bias that may result in under-reporting of sexual activity. The information was self-reported and it may not indicate the true picture of contraceptive practice by an adolescent. The data are from a cross-sectional survey and unable to establish any causal relationship between a response variable (contraceptive use) and the covariates of interest.

## Conclusion and recommendation

There is an increment in the trend of contraceptive use among sexually active female adolescents in Ethiopia between 2000 to 2016. A change in the pattern of long-acting and reversible contraceptive methods was observed. Personal, socioeconomic (macro), and health care system (intermediate) factors determine the contraceptive practices among sexually adolescent girls in Ethiopia.

Governments must develop more targeted strategies for improving adolescent's socioeconomic and education status. This will not only increase contraceptive prevalence, but will also reduce teenage pregnancy and birth, and in turn, contribute to the achievement of the Sustainable Development Goal 3 of good health and well-being. Improving contraception use among sexually active adolescents will also require connecting adolescents with information and services during their routine health service visits and taking advantage of missed opportunities for contact with the health facility.

## Data Availability

The datasets used and/or analyzed during the current study are available from the corresponding author on reasonable request. The raw data available at https://dhsprogram.com/publications/publication-fr328-dhs-final-reports.cfm.

## Abbreviations

AOR: Adjusted odd ratio
COR: Crude odd ratio
DHS: Demographic and Health Surveys
EAs: Enumeration areas
EDHS: Ethiopian Demographic and Health Surveys
HIV/AIDS: Human immunodeficiency virus infection and acquired immune deficiency syndrome
IUCD: Intra-Uterine Contraceptive Device
SPSS: Statistical Package for the Social Sciences
STIs: Sexual transmitted infections
TV: Television
